# The Gut Microbiome of Preterm Infants Treated With Aminophylline Is Closely Related to the Occurrence of Feeding Intolerance and the Weight Gain

**DOI:** 10.3389/fnut.2022.905839

**Published:** 2022-05-27

**Authors:** Wei Shen, Wen Qiu, Qi Lin, Chao Zeng, Yuting Liu, Weimin Huang, Hongwei Zhou

**Affiliations:** ^1^Microbiome Medicine Center, Department of Laboratory Medicine, Zhujiang Hospital, Southern Medical University, Guangzhou, China; ^2^Department of Neonatology, Nanfang Hospital, Southern Medical University, Guangzhou, China

**Keywords:** aminophylline, gut microbiome, feeding intolerance, preterm infants, weight gain, *Streptococcus*, *Rothia*

## Abstract

**Background:**

Aminophylline is widely used in the treatment of preterm infants, but it can cause feeding intolerance events, in which gut microbial dysbiosis may have a role. This study aims to investigate the relationship between the gut microbiome of preterm infants treated with aminophylline and the occurrence of feeding intolerance and weight gain rate.

**Methods:**

This study included a cohort of 118 preterm infants. Survival analysis and multivariate Cox regression were used to evaluate the relationship between aminophylline treatment and the occurrence of feeding intolerance. 16S rRNA V4 region gene sequencing was used to characterize the microbiome of fecal samples from the cohort. Linear discriminant analysis effect size was used to analyze the differential abundance of bacteria related to aminophylline treatment. Wilcoxon test, Kruskal–Wallis test, Spearman correlation coefficients and generalized linear mixed models were used to analyze the correlation between the differential bacteria and feeding intolerance events as well as the weight gain.

**Results:**

The results showed that the use of aminophylline could significantly increase the occurrence of feeding intolerance. The relative abundances of *Streptococcus* and *Rothia* in the gut microbiome of preterm infants were positively correlated with both the occurrence of feeding intolerance and the use of aminophylline, while the relative abundance of *Staphylococcus* was negatively correlated. In particular, preterm infants with a lower relative abundance of *Rothia* were more likely to develop feeding intolerance associated with aminophylline, and this difference existed before the onset of feeding intolerance. Moreover, it took longer for individuals with a lower relative abundance of *Streptococcus* to reach 2 kg weight. The contribution of *Streptococcus* to weight gain was greater than that of *Bifidobacterium* or *Lactobacillus*.

**Conclusion:**

The gut microbiome in preterm infants treated with aminophylline was characterized by a decrease in *Streptococcus* and *Rothia* and an increase in *Staphylococcus*. These microbes, especially *Rothia*, were positively correlated with the occurrence of feeding intolerance. *Streptococcus* but not *Bifidobacter* likely participated in the weight gain of preterm infants in early life.

## Introduction

Aminophylline is widely used in the treatment of critically ill neonates and preterm infants to stimulate the respiratory center, prevent renal injury after hypoxia and ischemia, and relax pulmonary vascular smooth muscle (1, 2). Similar to adults, neonates will experience increased heart rate, increased blood sugar, and increased urine output during the use of aminophylline, and these symptoms are usually mild. However, in the course of clinical application, it was found that some neonates had related gastrointestinal reactions to aminophylline, accompanied by the occurrence of feeding intolerance (FI) events, manifested as vomiting, abdominal distension, gastric retention, and even bloody stools after enteral feeding (3, 4). FI is an important event that affects early enteral nutrition in preterm infants, leading to a prolonged time to full enteral feeding, the occurrence of extrauterine growth retardation, and an increase in parenteral nutrition time (4, 5); FI is also the early manifestation of some severe digestive tract diseases and systemic infections, such as necrotizing enterocolitis and neonatal sepsis (6). The prevention and treatment of FI are of great significance in improving the survival rate of preterm infants.

As a factor closely related to the health of the human digestive tract, the gut microbiome has been reported in recent years to play an important role in early life (7), participate in the occurrence and development of many neonatal diseases, and serve as the key factor in growth and nutrition (8). Preterm infants experience a number of unique challenges to the establishment of their microbiota, the medicine of maternal and neonatal exposure, birth gestational, and the sterile environment of the neonatal intensive care unit (NICU) may all alter the microbiome. The gut microbiome of preterm infants in early life is dominated by opportunistic pathogens (such as *Staphylococcus, Streptococcus*, and *Enterobacter*), and the abundance of *Bifidobacterium* is low. The gut microbiome of preterm infants in this period follows a specific rule (9, 10), under which it is most closely related to age after birth (11) and is greatly influenced by postnatal diseases and clinical intervention (10, 12–14).

Based on the abovementioned clinical phenomena and studies, we hypothesize that the use of aminophylline in early life is associated with specific changes in the gut microbiome and that this association might be related to the occurrence of FI and even to the growth, development and nutrient absorption of preterm infants in early life. Therefore, we designed a prospective observational cohort study, combined with a nested case-control model, to focus on the gut microbiome of preterm infants related to aminophylline treatment for the first time and elucidate its relationship with FI events and early weight gain. The results provide clues for further mechanistic studies.

## Materials and Methods

### Study Participants and Experimental Design

This study was based on a prospective observational cohort of preterm infants in the NICU of Nanfang Hospital of Southern Medical University. Inclusion criteria included (a) gestational age (GA) <35 weeks and birth weight <2000 g; (b) a transfer to the NICU after birth and survival for >72 hours; and (c) informed consent of family members. The exclusion criteria included the following: (a) patients with digestive tract malformations and inherited metabolic diseases; (b) patients who used methylxanthine drugs other than aminophylline; and (c) patients whose families refused treatment or who had not reached full enteral feeding when transferred to another hospital due to other diseases. According to whether aminophylline was used, the cohort was divided into two groups for comparative analysis. The usage of aminophylline and the age at the time of the FI incident was investigated, and clinical data such as GA, weight, sex mode of delivery, time to full enteral feeding and daily weight were collected. Subjects were matched for the nested case-control design according to the needs of the analysis. The diagnostic criteria for FI are as follows: (a) gastric residual volume exceeding 50% of the previous feeding amount, accompanied by vomiting and/or abdominal distension; or (b) failure of the feeding plan, including a reduction, delay or interruption of enteral feeding (15).

The research was reviewed and approved by the Ethics Committee of Nanfang Hospital of Southern Medical University (NFEC-2021-054). The study was enrolled in the Chinese Clinical Trial Register (trial registration No. ChiCTR2100044469).

### Sample Collection and 16S Ribosomal RNA Gene Sequencing

Postnatal fecal samples were collected from cohort members 1–2 times per week until discharge. After being collected from diapers, the fresh fecal samples were stored at −40°C in time, transported back to the laboratory within 3 days, and then stored at −80°C. According to the instructions, the QIAamp Mini Kit was uniformly used to extract DNA from the fecal samples. The V4 hypervariable region of the 16S ribosomal RNA (rRNA) gene was amplified and sequenced using an Illumina iSeq 100 platform.

### Processing of Sequence Data

The raw fastq sequence data were demultiplexed into separate paired-end fastq files based on the barcodes using in-house Perl scripts. Amplicon sequence variants (ASVs) were obtained by denoising each sample paired-end fastq file using the R package DADA2 (16) (version 1.6.0). The PyNAST (17) algorithm was used to align representative sequences, and FastTree (18) was used to build a phylogenetic tree. Taxonomic classification was performed with the Ribosomal Database Project classifier (19) against the GreenGenes database (20) (version 13.8). The ASVs from reagent controls were excluded from downstream analysis. Each sample was rarefied to 3,180 sequences, and those samples with fewer than 3,180 sequences were discarded from downstream analysis.

### Biostatistical Analysis

All subsequent biostatistical analyses were conducted using QIIME (21) (version 1.9.1) software or the R language (version 4.1.0). Kaplan–Meier survival curves were generated and compared by the log-rank test. Multivariate Cox regression analysis was performed to identify significant factors associated with FI. The Shannon index and phylogenetic diversity (PD) whole-tree index were calculated to estimate the α-diversity of the gut microbiome. Assessment of bacterial compositional changes was performed using the MaAsLin2 (22) (version 1.6.0) R package and linear discriminant analysis (LDA) effect size (LEfSe) (23). To better show the relative abundance distribution of bacteria in the figure, the relative abundances were arcsine square root transformed. Two nested case-control cohorts were obtained by 1:1 propensity score matching (PSM) to reduce some common confounding biases. To estimate the effect sizes of different variables, a multivariate generalized linear mixed model (GLMM) was fitted by taking groups with weekly weight gain rate (weekly_WGRs)≥ 0.06 as the dependent variable, some variables of interest as fixed effects, and patients as a random effect in the R package ‘lme4' (version 1.1–27.1).

For comparisons of categorical data, the chi-square test or Fisher's exact test was performed depending on the theoretical frequency. The Wilcoxon rank-sum test and the Kruskal- Wallis test, respectively, were compared of distributions between two groups or between two groups. Correlations between two variables were estimated with Spearman correlation coefficients. The Benjamini–Hochberg false discovery rate (FDR) method was adopted to adjust for multiple comparisons. For all comparisons, a *P*-value or FDR-corrected *P*-value <0.05 was considered statistically significant.

## Results

### Characterization of the Cohort

**Figure 1:**
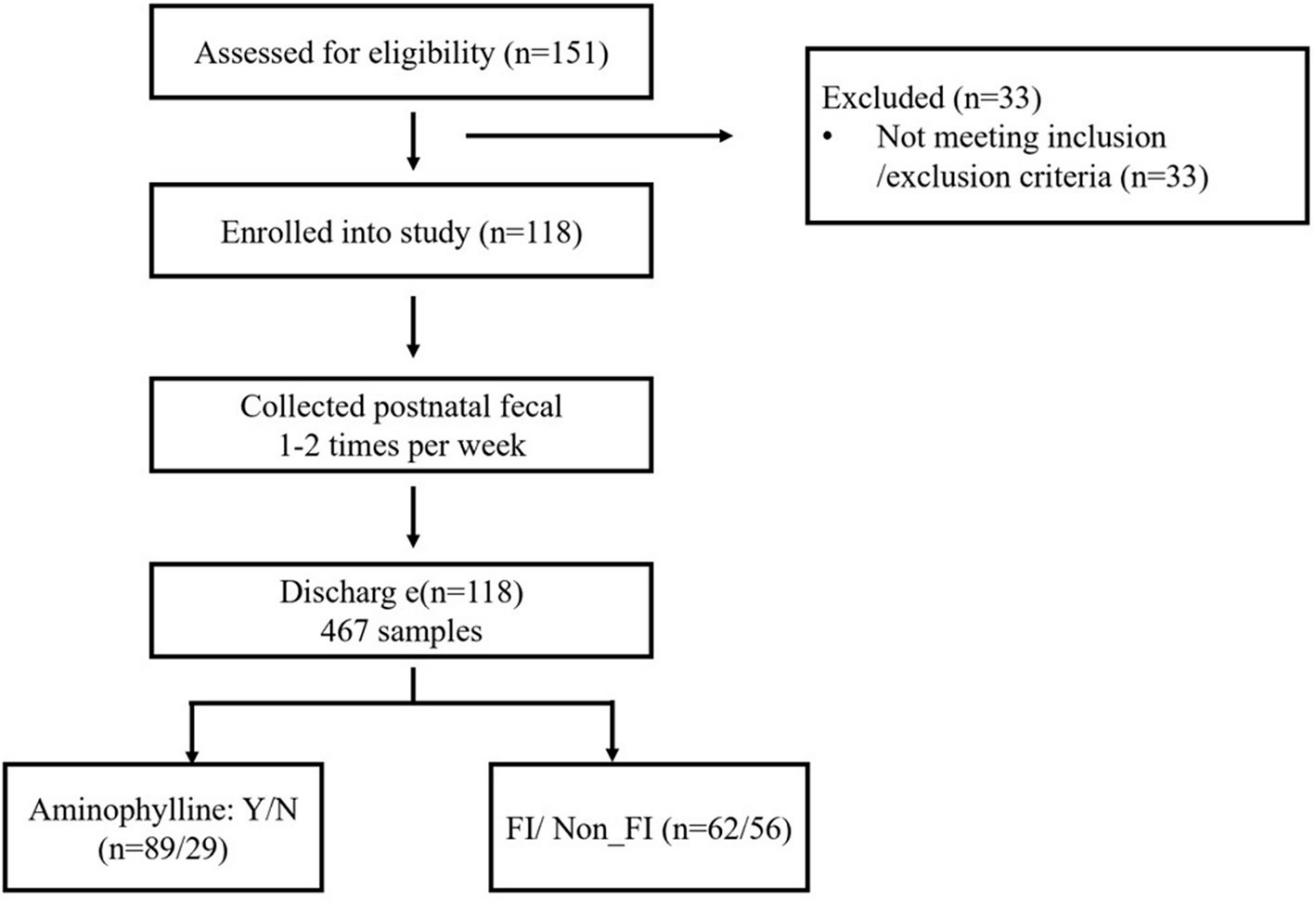
Study flow diagram.

During the recruitment period, 151 infants from the prospective cohort of preterm infants at Nanfang Hospital of Southern Medical University were screened, and 118 infants enrolled into the study (Figure 1). The recruited infants had an average GA of 31.2±2.5 weeks, an average birth weight of 1.49 ± 0.41 kg, an aminophylline usage rate of 75.42, and a 52.54% incidence of FI (Table 1). A total of 467 fecal samples were collected, and 6,013,140 high-quality sequences were obtained after iSeq sequencing and quality control. The median number of sequences contained in each sample was 11,991 (3,526–43742); after the data were denoised with DADA2, 8086 ASVs were obtained. Next, the samples were flattened to 3,500 sequences, and samples from patients with unknown FI onset times were removed; 7,507 ASVs that remained after these steps were included in the downstream analysis.

**Table 1:** Characterization of the cohort.

**Variables**	**Original cohort**	**Aminophylline nested case–control cohort**		**FI nested case–control case cohort**	
		**Aminophylline (*N* = 29)**	**Non-aminophylline (*N* = 29)**	**FI (*N* = 30)**	**Non-FI (*N* = 30)**
Sample size	467	80	86	148	105
Gestational age (weeks)	31.2 ± 2.5	32.9 ± 1.8	33.2 ± 1.8	30.3 ± 2.2	31.0 ± 2.0
Birth weight (kg)	1.49 ± 0.41	1.69 ± 0.38	1.80 ± 0.41	1.42 ± 0.45	1.50 ± 0.32
Sex, *N* (%)					
Female	37 (31.36)	13 (22.41)	11 (18.97)	8 (13.33)	8 (13.33)
Male	81 (68.64)	16 (27.59)	18 (31.03)	22 (36.67)	22 (36.67)
Aminophylline, *N* (%)					
Y	89 (75.42)	29 (100)	0 (0)	25 (41.67)	23 (38.33)
N	29 (24.58)	0 (0)	29 (100)	5 (8.33)	7 (11.67)
Course of aminophylline (days)	21.7 ± 14.7	13.1 ± 10.1	NA	19.5 ± 19.3	18.4 ± 18.3
FI, N (%)					
Yes	62 (52.54)	14 (24.14)	5 (8.62)	30 (100)	0 (0)
No	56 (47.46)	15 (25.86)	24 (41.38)	0 (0)	30 (100)
FI age^#^ (days)	9.0 ± 7.2	8.6 ± 8.2	10.4 ± 10.6	8.7 ± 7.5	NA
FEF_time in FI patient (days)	22.3 ± 14.2	15.4 ± 8.6	19.4 ± 11.3	22.2 ± 16.2	NA
Age when weight reached 2 kg(days)	27.89 ± 17.36	18.2 ± 13.2	13.7 ± 13.4	33.2 ± 21.9	24.2 ± 14.4

# *The age at the time of the FI incident was investigated.*

### The Incidence of FI in Preterm Infants Treated With Aminophylline Was Significantly Higher Than That in the Control Group

**Figure 2:**
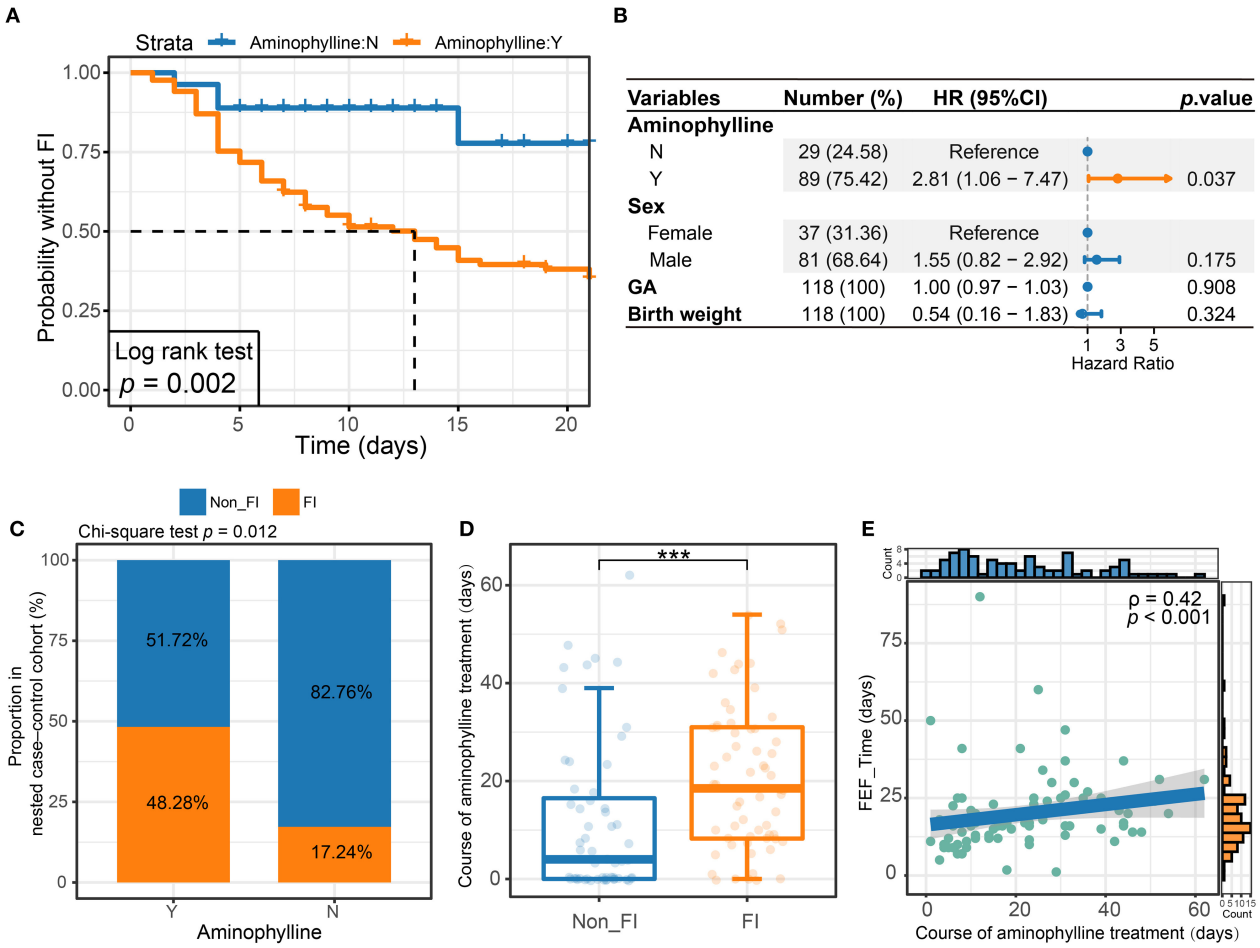
The relationship between aminophylline treatment and FI events. **(A)** Kaplan–Meier survival analysis of FI between groups treated with aminophylline. The group treated with aminophylline had a higher FI risk (log rank *P* = 0.002). **(B)** Forest plot showing the results of multivariate Cox regression analyses. The points and horizontal lines correspond to the adjusted hazard ratios and 95% confidence intervals. Orange indicates statistical significance (*P* < 0.05). **(C)** Stacked bar plot showing that the proportions of FI were different between groups treated with aminophylline in the aminophylline nested case-control cohort. **(D)** Boxplot showing the course of aminophylline in patients with FI and without FI. ***, *P* < 0.001. **(E)** Scatter plot showing the correlation between time to full enteral feeding (FEF_Time) and the course of aminophylline in patients taking aminophylline. The histograms above and on the right show the distribution of the course of aminophylline and FEF_Time, respectively. Spearman correlation coefficients (ρ values) and *P*-values are shown in the top left corner.

To evaluate the effect of aminophylline treatment on FI events in this study, we used Kaplan–Meier survival analysis to compare aminophylline treatment during the most frequent period of FI events (the first 3 weeks after birth). The results showed that the risk of FI events in the aminophylline treatment group (aminophylline: Y) was significantly increased (Figure 2A). This difference still existed after controlling for factors such as GA, gender and birth weight of the cohort (Figure 2B, *P* = 0.037). According to whether aminophylline was used, we further established a small nested case-control cohort by PSM of the whole cohort. The nested case-control cohort for aminophylline included a total of 166 samples, collected from 29 patients in the treatment group and 29 patients in the control group. There were no significant differences in GA, birth weight, or sex between the two groups in the aminophylline nested case-control cohort (Table 1). The incidence of FI in the aminophylline treatment group from the nested case-control cohort was 48.28%, while that in the control group was 17.24%, and the difference was still statistically significant (Figure 2C, *P* =0.012). When the course of aminophylline treatment was further analyzed across all aminophylline-treated populations, it was found that preterm infants with FI events had a longer course of aminophylline than preterm infants who did not (Figure 2D, *P* < 0.001), and the length of the course was positively correlated with the time to reach full enteral feeding in preterm infants (Figure 2E, ρ = 0.42, *P* < 0.001).

### Preterm Infants Treated With Aminophylline Showed Characteristic Changes in the Gut Microbiome, Which Were Related to FI

**Figure 3:**
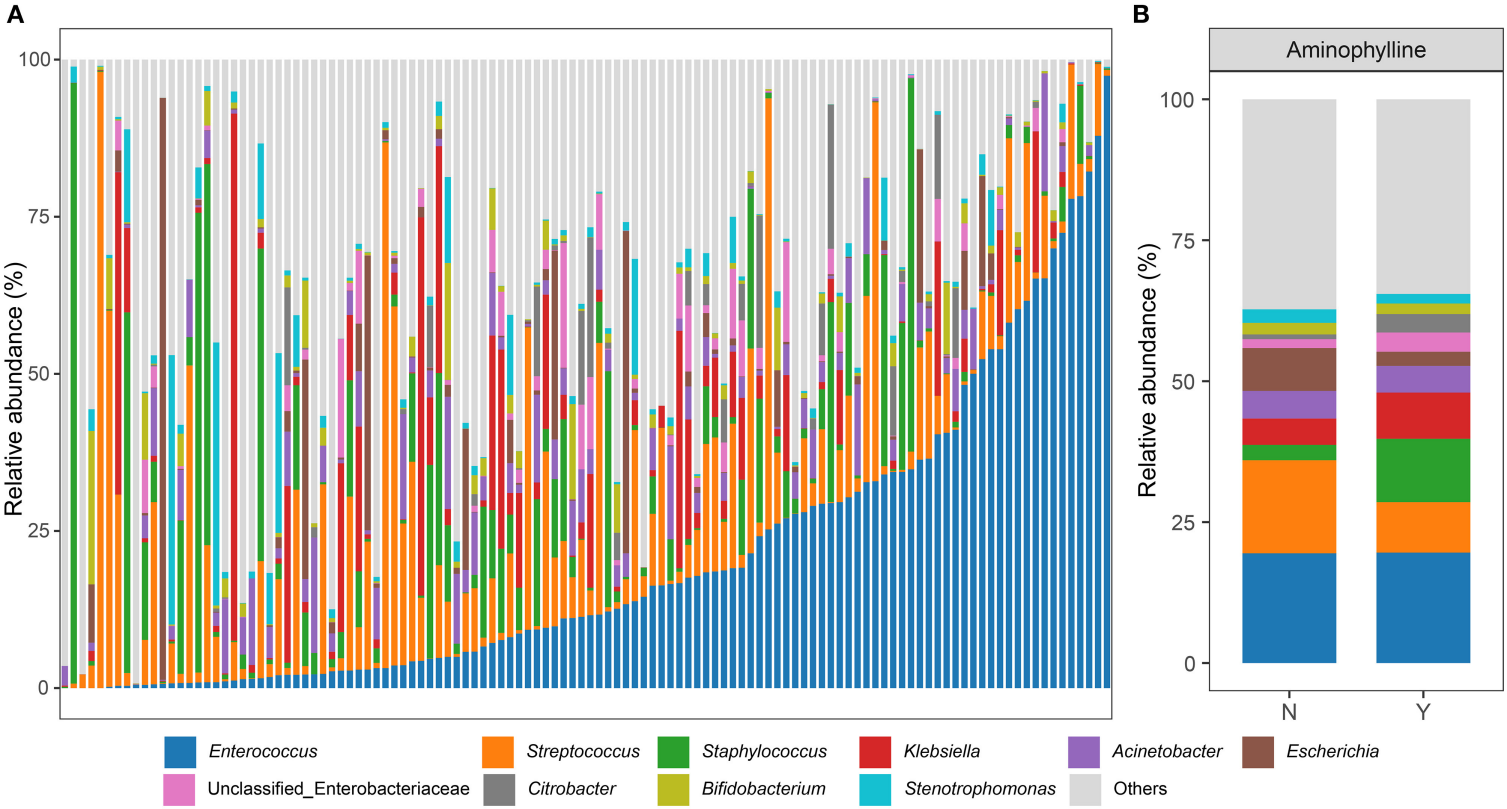
Genus-level microbiome composition in patients. **(A)** Relative abundances of the top 10 abundant genera are displayed. Each bar represents the mean abundance of all samples of a patient. **(B)** The relative abundances at the genus level between patients who received aminophylline and those who did not.

We sequenced and identified the fecal samples of the preterm infants after birth by 16S rRNA sequencing. The gut microbiome of preterm infants in this study was mainly composed of Firmicutes and Proteobacteria, among which the bacterial genera with the highest relative abundance were *Enterococcus, Streptococcus, Staphylococcus, Klebsiella* and *Acinetobacter*. However, *Bifidobacterium, Escherichia* and other genera had low relative abundance, and a few individuals were characterized by an absolute dominance of a single genus of gut microbes (Figure 3A). When the cohort was divided according to the use or non-use of aminophylline, it was found that the relative abundances of *Streptococcus, Staphylococcus, Klebsiella* and *Escherichia* in the two groups differed (Figure 3B).

**Figure 4:**
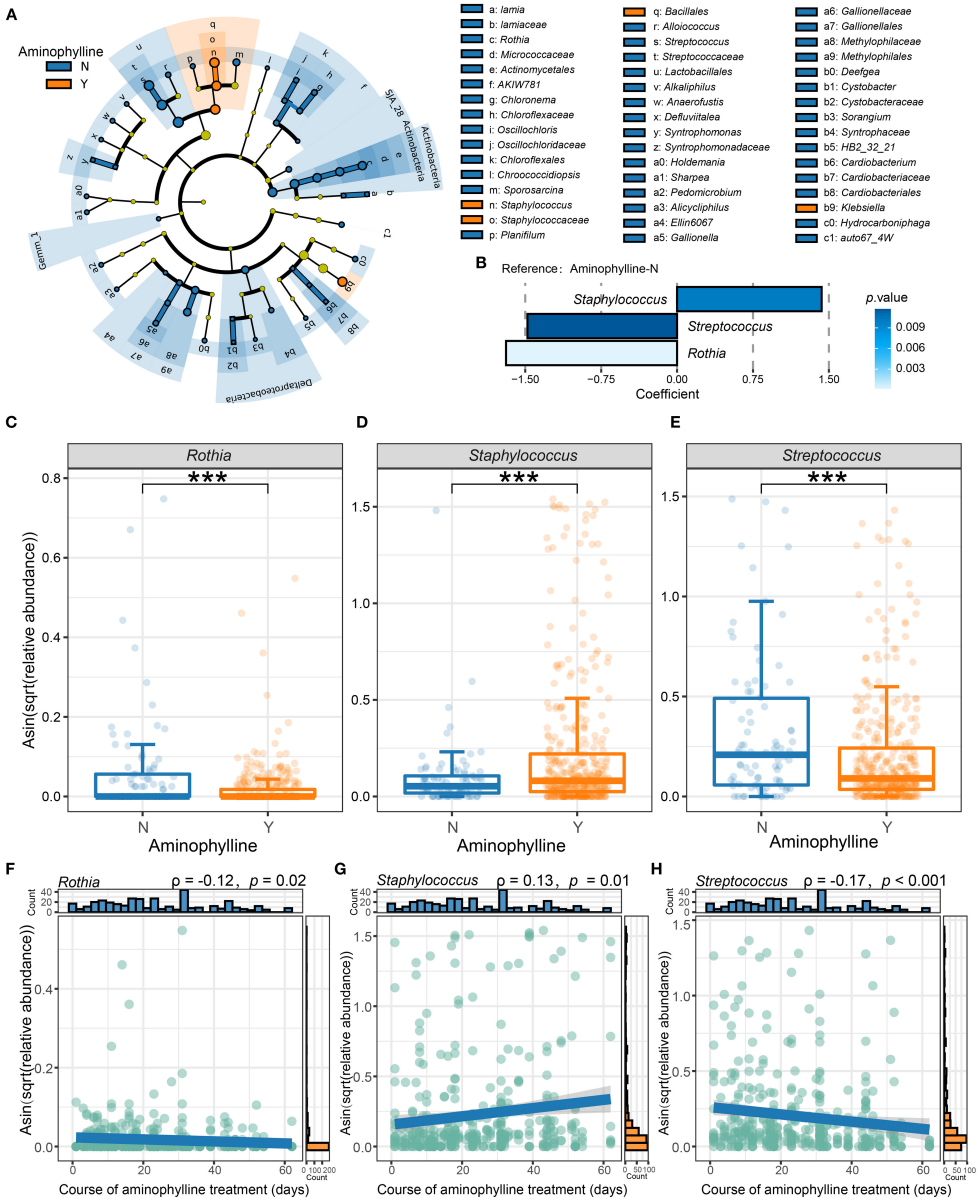
Differences in the microbiome between the aminophylline-treated group and the control group. **(A)** A cladogram of the LEfSe analysis shows the significantly differentially abundant bacteria between the group that received aminophylline and the group that did not. Taxonomic levels are represented by rings with phyla in the innermost ring and genera in the outermost ring. The significantly different taxa are colored by different colors representing the two groups (LDA score >2), and the yellow dots represent the taxa without significant differences. **(B)** The changes in the relative abundance of genera between the groups with and without aminophylline treatment, estimated using MaAsLin2 by taking the non-aminophylline group as a reference. Each bar represents the coefficient estimated by MaAsLin2, and the depth of the color represents the *P*-value (only genera with a *P*-value <0.05 are displayed). **(C**–**E)** Boxplots and jittered points show the abundance distribution of *Rothia* **(C)**, *Staphylococcus* **(D)** and *Streptococcus* **(E)** between groups treated with aminophylline. Each point represents a sample. ^***^, *P* < 0.001. **(F–H)** Scatter plots showing the correlation between the abundances of *Rothia* **(F)**, *Staphylococcus* **(G)** and *Streptococcus* **(H)** and the course of aminophylline in patients taking aminophylline. The histograms above and on the right show the distribution of the course of aminophylline and abundances, respectively. Spearman correlation coefficients (ρvalues) and *P*-values are shown in the top left corner.

To further clarify whether there was a significant difference in the microbiome between the aminophylline treatment group and the control group, we conducted LEfSe analysis, compared the microbiome composition of the two groups at the genus level, and estimated the change in the relative abundance of microbes at the genus level combined with MaAsLin2 (FDR < 0.25) (Figures 4A,B). The results showed that *Rothia* (β = −1.689675, *P* = 0.000159), *Staphylococcus* (β = 1.428663, *P* = 0.010141) and *Streptococcus* (β = −1.478244, *P* = 0.011750) had significant differences in relative abundance between the gut microbiomes of the two groups. Therefore, we focused on the relationship between these three bacteria and aminophylline treatment. Using the Wilcoxon test, we found that the relative abundances of *Rothia* (*P* < 0.001) (Figure 4C) and *Streptococcus* (*P* < 0.001) (Figure 4D) in the treatment group decreased significantly; however, *Staphylococcus* (*P* < 0.001) (Figure 4E) increased significantly, and these differences were still significant after considering the influence of postmenstrual age on the evolution of the microbiome in the GLMM (*Rothia, P* = 0.007; *Streptococcus, P* = 0.002; *Staphylococcus, P* = 0.0455). At the same time, the relative abundances of *Rothia* (ρ =-0.12, *P* = 0.02) and *Streptococcus* (ρ = 0.13, *P* = 0.01) in the gut microbiome of preterm infants who took aminophylline were negatively correlated with the course of treatment, while *Staphylococcus* (ρ =-0.17, *P* < 0.001) was positively correlated with it (Figures 4F–H).

**Figure 5:**
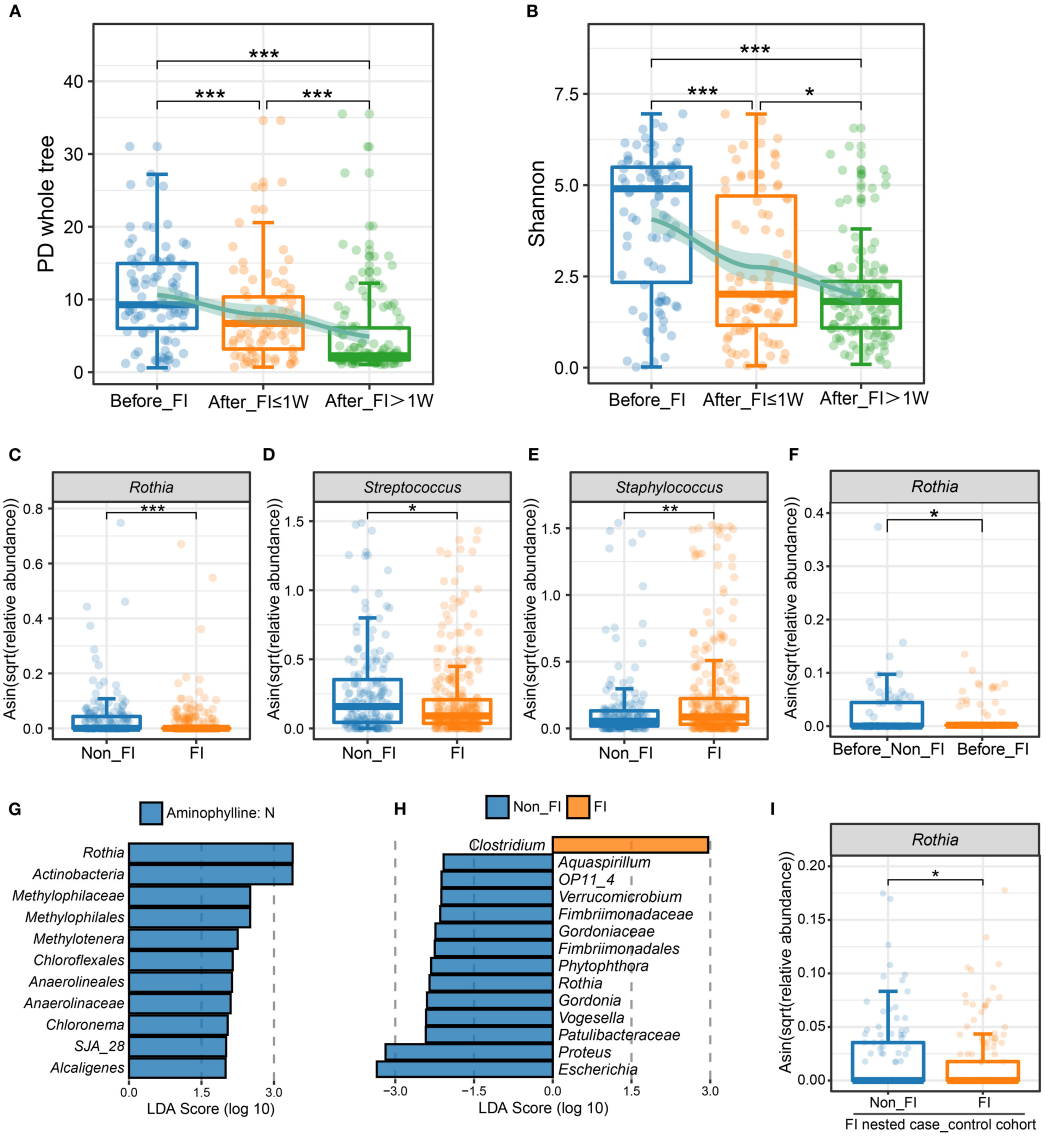
Differences in the microbiome between patients with FI and without FI. **(A,B)** Comparison of the PD whole-tree index **(A)** and Shannon index **(B)** among different FI stage groups. The jittered scatter plot represents the α-diversity index of each sample. The curves on the top layers of the graphs were fitted with the LOESS method. *, FDR < 0.05; **, FDR < 0.01; ***, FDR < 0.001. **(C**–**E)** Boxplots and jittered points show the abundance distribution of *Rothia* **(C)**, *Streptococcus* **(D)** and *Staphylococcus* **(E)** between the non-FI and FI groups. Each point represents a sample. *, *P* < 0.05; **, *P* < 0.01; ***: *P* < 0.001. **(F)** Boxplot and jittered points show the abundance distribution of *Rothia* between the before-FI (samples before the FI onset) and before-non-FI (samples before 6 days age) groups. Each point represents a sample. *, *P* < 0.05. **(G)** Significantly differentially abundant bacterial taxa between FI patients with and without aminophylline, estimated by LEfSe. Each bar represents the LDA score for a specific taxon. The color of a bar indicates that the taxon was enriched in the corresponding group. **(H)** Significantly different bacterial taxa between the non-FI and FI groups among patients treated with aminophylline. **(I)** Comparison of *Rothia* abundance between the non-FI and FI groups in the FI nested case-control cohort. *, *P* < 0.05.

In addition to finding that the characteristics of the microbiome were related to the treatment of aminophylline, we also found that when FI occurred in preterm infants, the microbiome also showed certain predictable characteristics. The α-diversity (PD whole tree, Shannon) of this group of preterm infants gradually decreased in the following three stages: before onset, within 1 week after onset, and 1 week after onset (Figures 5A,B), and the differences among groups were statistically significant, indicating that there were significant changes in the microbiome before and after the onset of FI. To explore whether these changes were associated with aminophylline-related microbiome characteristics, we also compared three aminophylline-related bacteria between the FI group and the non-FI group. The results showed that the relative abundances of *Rothia* (*P* < 0.001) and *Streptococcus* (*P* = 0.02307) also showed a significant downward trend in the FI group, while that of *Staphylococcus* (*P* = 0.007105) showed a significant increase (Figures 5C–E). These characteristics are consistent with the changes in three bacteria in the aminophylline treatment group. When a further comparison was made between the samples before the onset in the FI group and those before the corresponding age (taking the median age of 6 days in the FI group) in the non-FI group, it was found that the relative abundance of *Rothia* decreased significantly before the onset of FI (Figure 5F, *P* = 0.04576).

Considering the various causes of FI events, to further explore the other bacteria that were involved in FI events related to aminophylline treatment, we also compared the gut microbiomes of all preterm infants with FI events according to whether aminophylline was used, intending to explore the microbiomic characteristics that correspond to FI events related to aminophylline (Figure 5G). At the same time, the gut microbiomes of preterm infants with FI events and those without FI events were compared among all the preterm infants that were treated with aminophylline, screening for bacteria associated with both aminophylline treatment and FI incidence from another perspective (Figure 5H). The results of the two comparisons suggested that the common element was *Rothia*.

As many basic features of preterm infants may affect the onset of FI, we attempted to further confirm whether the reduced relative abundance of *Rothia* was an important factor that influenced the occurrence of FI in aminophylline users. In this prospective cohort, we adopted the PSM method, according to whether FI occurred, and matched a group of nested case-control cohorts to the greatest extent for verification (Table 1). A total of 253 samples were included in this small cohort, with the disease group and the control group containing 30 cases each. Aminophylline use was consistent between the two groups, and there was no significant difference in GA, birth weight, or gender. Comparing the differences in *Rothia* between the two groups again, the results showed that the abundance of *Rothia* in the FI group after matching was still lower, and this difference was unrelated to GA and body weight (Figure 5I, *P* = 0.04978).

### The Relationship Between Aminophylline-Related Microbiome Features and the Rate of Weight Gain in Preterm Infants

Since (i) both aminophylline treatment and an associated FI event may affect weight gain in preterm infants, and (ii) the results discussed in previous sections suggested that aminophylline treatment was strongly associated with the characteristics of the microbiome, we further investigated weight gain during hospitalization of all preterm infants in this cohort. The age (day of life) when the bodyweight reached 2 kg and the weekly_WGR were used as indicators to evaluate the relationship between some bacteria and the weight gain of preterm infants.

**Figure 6:**
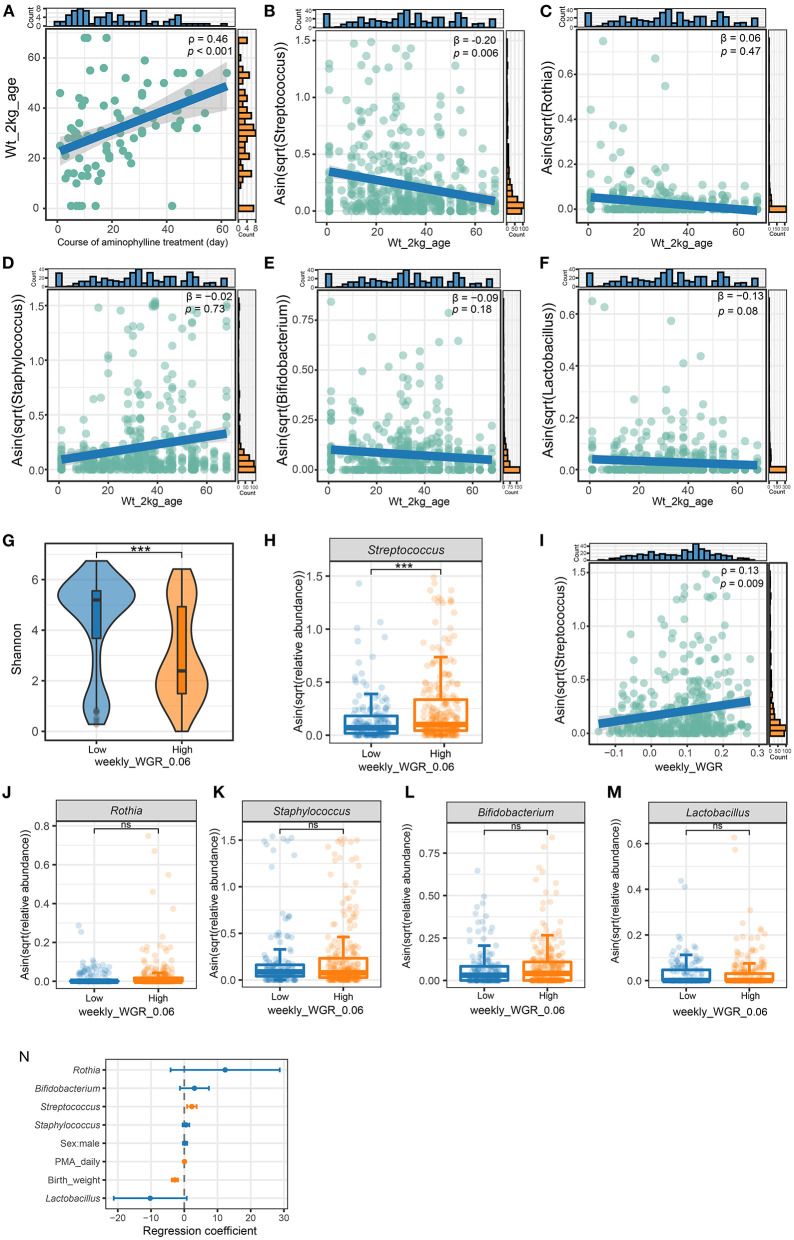
The relationship of weight gain with microbiome and aminophylline. **(A)** The correlation between age in days when the patient's weight reached two kilograms and the course of aminophylline in patients taking aminophylline. **(B**–**F)** The correlation between age in days when the patient's weight reached two kilograms and the abundance of *Streptococcus* **(B)**, *Rothia* **(C)**, *Staphylococcus* **(D)**, *Bifidobacterium* **(E)** and *Lactobacillus* **(F)**. β is the regression coefficient for *Streptococcus* and age in days when the patient's weight reached two kilograms after adjusting covariates (gestational age, sex, and birth weight). **(G)** Comparison of the Shannon index between groups with weekly_WGR ≥ 0.06. **(H)** Comparison of the abundances of *Streptococcus*. ***: *P* < 0.001. **(I)** The correlation between *Streptococcus* and weekly_WGR for each sample collected. **(J**–**M)** Comparison of the abundances of *Rothia* **(J)**, *Staphylococcus* **(K)**, *Bifidobacterium* **(L)** and *Lactobacillus* **(M)** between groups with weekly_WGR ≥ 0.06. ***, *P* < 0.001; ns, no statistical significance. **(N)** multivariate GLMM plots to visualize the model result, showing the effect size for each independent variable by taking groups with weekly_WGR ≥ 0.06 as the dependent variable. The points and horizontal lines correspond to the adjusted GLMM coefficients and 95% confidence intervals. Orange significant indicates statistical significance (*P* < 0.05).

First, we counted the age at which each preterm infant in the cohort reached a bodyweight of 2 kg. If an infant's weight had not reached 2 kg at the time of discharge, the weight gain would be estimated to be 15 g/kg per day. This indicator reflects the speed of weight gain to a certain extent. Statistically, among preterm infants receiving aminophylline, as the duration of medication increased, the age at which the preterm infants reached a weight of 2 kg increased correspondingly (Figure 6A, ρ = 0.46, *P* < 0.001). To explore whether this effect was related to the characteristics of the microbiome after aminophylline treatment, we performed GLMM with age at 2 kg as the dependent variable and the relative abundance of bacteria, birth weight, GA, and sex as independent variables; we found that the relative abundance of *Streptococcus* in the gut microbiome of preterm infants was negatively correlated with their age reaching a weight of 2 kg after excluding the influence of other factors (Figure 6B, β = 0.2, *P* = 0.006), while *Rothia* (Figure 6C), β = 0.06, *P* = 0.47), *Staphylococcus* (Figure 6D, β =-0.02, *P* = 0.73), *Bifidobacterium* (Figure 6E, β =-0.09, *P* = 0.18) and *Lactobacillus* (Figure 6F, β =-0.13, *P* = 0.08) were not significantly different.

To clarify the relationship between weight gain and *Streptococcus*, we further recorded the ages of preterm infants in weeks at the times when 437 fecal samples were collected from the cohort, and we cross-referenced the samples with the weekly_WGR of preterm infants at this age. Then, according to whether the corresponding weekly_WGR was lower than 6% (converted from the standard of daily weight gain <10 g/kg), all samples were divided into high and low groups. The results of microbiome analysis showed that there were significant differences in α-diversity between these two groups (Figure 6G). The faster the weekly_WGR, the lower the diversity (Shannon, *P* = 2.13e−08). The relative abundance of *Streptococcus* was significantly different between the two types of samples (Figure 6H, *P* < 0.001). Regardless of whether the samples came from the same preterm infant, the relative abundance of *Streptococcus* was lower in the samples with a slower weekly_WGR (low group), and *Streptococcus* was positively correlated with weekly_WGR (Figure 6I, ρ = 0.13, *P* = 0.009). Similarly, the relative abundances of *Rothia* (*P* = 0.54), *Staphylococcus* (*P* = 0.87), *Bifidobacterium* (*P* = 0.07) and *Lactobacillus* (*P* = 0.24) were not significantly different between the two groups (Figures 6J–M). Taking a weekly_WGR ≥ 0.06 as the dependent variable and including birth weight, postmenstrual age, gender and other common factors that would affect weight as independent variables, we constructed a GLMM to show the degree of influence of each factor on the weekly_WGR. The model showed that the contribution of *Streptococcus*'s relative abundance to weekly_WGR ≥ 6% was second only to that of postmenstrual age and greater than that of birth weight, with no corresponding contribution from other bacteria (Figure 6N).

## Discussion

In recent years, many studies have reported that specific drugs may affect the gut microbiome (24) and may also affect the host through microbial metabolism (25–27). In the course of studying this cohort of 118 preterm infants, we found the following interesting links between aminophylline, FI events, and the gut microbiome.

First, the preterm infants treated with aminophylline not only had a significantly higher incidence of FI events (Figure 2) but also exhibited characteristic changes in the gut microbiome (Figures 3, 4). Aminophylline is a type of methylxanthine drug that acts as a nonselective adenosine receptor antagonist to provide renal protection in hypoxic-ischemic conditions (28) and is also often used to prevent apnea in preterm infants by inhibiting phosphodiesterase (1, 29). Adverse gastrointestinal reactions to aminophylline have been reported frequently (3, 4), but no researchers have investigated whether the drug leads to changes in the gut microbiome. In this study, the duration of aminophylline use was found not only to be associated with an increased incidence of FI in preterm infants (Figures 2A–D) but also to delay enteral nutrition in preterm infants (Figure 2E). Compared with the control group, the gut microbiome of preterm infants treated with aminophylline was characterized by lower relative abundances of *Rothia* and *Streptococcus* and a higher relative abundance of *Staphylococcus* (Figures 4C–E). Moreover, this trend became more pronounced as the duration of the medication course increased (Figures 4F–H).

Second, the three bacteria also showed the same regularity in the FI onset population as in the aminophylline-treated population, especially *Rothia*, which was closely associated with aminophylline-related FI events (Figures 5G,H). The analysis showed that even in preterm infants with similar aminophylline use, FI was more likely to occur in those with a lower relative abundance of *Rothia* (Figure 5I), and this difference existed before the onset of FI (Figure 5F). *Rothia*, a butyrate-producing bacterium, has been found to colonize the human oral cavity and is also part of the characteristic microbiome carried in the gut of vaginally delivered neonates (30). A cohort study reported that it is one of the most characteristic symbiotic bacteria associated with breast milk and the infant's gut (31); research in another cohort also showed that this genus may mediate a protective effect against asthma through the metabolite butyrate (32), reducing chronic airway inflammation (33). While breastfeeding was an effective method to reduce the incidence of FI (34–36), it was speculated that a low relative abundance of *Rothia* may be a disadvantage during feeding in the aminophylline-treated preterm infant population.

Finally, we also observed that *Streptococcus* but not *Bifidobacterium* was involved in weight gain in the early life of preterm infants (Figures 6B,E,F,N), while the diversity of the microbiome decreased with increasing weight gain (Figure 6G). The gut microbiome in early life is closely related to the host's energy balance (37, 38), while *Bifidobacterium* and *Lactobacillus* are predominant in the gut of healthy full-term newborns (39, 40), and they are considered to be related to growth and play an important role in early life (41–44). However, multiple studies have found that *Enterobacteriaceae, Enterococcus, Staphylococcus*, and *Streptococcus* are the main components of the gut microbiome of preterm infants in early life, (9, 11, 45), and our cohort did show that the relative abundances of these bacteria were high, while the relative abundances of *Bifidobacterium* and *Lactobacillus* were low (Figure 3). Interestingly, we found that individuals with a low relative abundance of *Streptococcus* took longer to reach a bodyweight of 2 kg, but each time they entered a stage of increased *Streptococcus* abundance, their rate of weight gain increased accordingly (Figures 6H,I). This is consistent with other researchers' observations of a positive correlation between *Streptococcus* and body mass index (46). However, *Bifidobacterium* and *Lactobacillus* were not only low in relative abundance but also unassociated with early weight gain in preterm infants (Figures 6E,F,L–N), which may be due to their low total abundance and inability to contribute to early growth. Although some species of *Streptococcus*, such as *Streptococcus agalactiae*, are high-risk factors for infection in preterm infants, *Streptococcus* itself is one of the common components of the microbiome in early life (47) and is also one of the core microbes from breast milk (31). The role of *Streptococcus* in the early growth of preterm infants merits further exploration.

Our study was not yet able to show a clear causal association among aminophylline treatment, gut microbiome, FI events, and weight gain; however, for the preterm infant population, our study demonstrated that *Streptococcus, Staphylococcus*, and especially *Rothia* were associated with aminophylline treatment and FI events. More importantly, we found that *Streptococcus* was strongly associated with weight gain in the early life of preterm infants. Despite observing these novel and interesting clues for the first time, our study has some limitations. First, the overall sample size was small. Second, we merely identified associations among changes in the microbiome, aminophylline treatment, associated FI events and weight gain; whether there is truly a causal relationship is a topic that awaits further study in a larger cohort.

## Data Availability Statement

The datasets presented in this study can be found in online repositories. The names of the repository/repositories and accession number(s) can be found below: https://www.ncbi.nlm.nih.gov/, PRJNA803308.

## Ethics Statement

The studies involving human participants were reviewed and approved by the Ethics Committee of Nanfang Hospital of Southern Medical University. Written informed consent to participate in this study was provided by the participants' legal guardian/next of kin.

## Author Contributions

WS and HZ contributed to the conception and design of the study. WS, CZ, and YL collected all the materials. WS wrote the first draft of the manuscript. WQ and QL performed the statistical analysis and wrote sections of the manuscript. HZ and WH revised the manuscript. All authors contributed to the article and approved the submitted version.

## Funding

The work was supported by the National Natural Science Foundation of China (grant No. 81925062 to HZ). The funders had no role in the study design, data collection and analysis, decision to publish, or preparation of the manuscript.

## Conflict of Interest

The authors declare that the research was conducted in the absence of any commercial or financial relationships that could be construed as a potential conflict of interest.

## Publisher's Note

All claims expressed in this article are solely those of the authors and do not necessarily represent those of their affiliated organizations, or those of the publisher, the editors and the reviewers. Any product that may be evaluated in this article, or claim that may be made by its manufacturer, is not guaranteed or endorsed by the publisher.
